# Temporary Storage or Permanent Removal? The Division of Nitrogen between Biotic Assimilation and Denitrification in Stormwater Biofiltration Systems

**DOI:** 10.1371/journal.pone.0090890

**Published:** 2014-03-26

**Authors:** Emily G. I. Payne, Tim D. Fletcher, Douglas G. Russell, Michael R. Grace, Timothy R. Cavagnaro, Victor Evrard, Ana Deletic, Belinda E. Hatt, Perran L. M. Cook

**Affiliations:** 1 Monash Water for Liveability, Department of Civil Engineering, Monash University, Victoria, Australia; 2 Department of Resource Management and Geography, Melbourne School of Land and Environment, The University of Melbourne, Victoria, Australia; 3 Water Studies Centre, School of Chemistry, Monash University, Victoria, Australia; 4 School of Biological Sciences, Monash University, Victoria, Australia; University Paris South, France

## Abstract

The long-term efficacy of stormwater treatment systems requires continuous pollutant removal without substantial re-release. Hence, the division of incoming pollutants between temporary and permanent removal pathways is fundamental. This is pertinent to nitrogen, a critical water body pollutant, which on a broad level may be assimilated by plants or microbes and temporarily stored, or transformed by bacteria to gaseous forms and permanently lost via denitrification. Biofiltration systems have demonstrated effective removal of nitrogen from urban stormwater runoff, but to date studies have been limited to a ‘black-box’ approach. The lack of understanding on internal nitrogen processes constrains future design and threatens the reliability of long-term system performance. While nitrogen processes have been thoroughly studied in other environments, including wastewater treatment wetlands, biofiltration systems differ fundamentally in design and the composition and hydrology of stormwater inflows, with intermittent inundation and prolonged dry periods. Two mesocosm experiments were conducted to investigate biofilter nitrogen processes using the stable isotope tracer ^15^NO_3_^−^ (nitrate) over the course of one inflow event. The immediate partitioning of ^15^NO_3_^−^ between biotic assimilation and denitrification were investigated for a range of different inflow concentrations and plant species. Assimilation was the primary fate for NO_3_^−^ under typical stormwater concentrations (∼1–2 mg N/L), contributing an average 89–99% of ^15^NO_3_^−^ processing in biofilter columns containing the most effective plant species, while only 0–3% was denitrified and 0–8% remained in the pore water. Denitrification played a greater role for columns containing less effective species, processing up to 8% of ^15^NO_3_^−^, and increased further with nitrate loading. This study uniquely applied isotope tracing to biofiltration systems and revealed the dominance of assimilation in stormwater biofilters. The findings raise important questions about nitrogen release upon plant senescence, seasonally and in the long term, which have implications on the management and design of biofiltration systems.

## Introduction

The performance of stormwater biofilters (also known as bioretention systems or raingardens) has traditionally been expressed in terms of simple pollutant removal. Few studies consider the permanency of this removal, yet many processes in such systems may be better described as attenuation - when retention is only temporary and the pollutant is at some point re-released, either in its original or transformed state. The fate of a pollutant between temporary and permanent removal pathways is fundamental to long-term performance. Nitrate is a critical waterway pollutant with possible transformations in both of these categories – biotic assimilation provides temporary immobilization, or denitrification offers permanent removal in gaseous form. While nitrogen transformation and cycling processes have been characterised across wide natural and engineered environments, they have not been explicitly quantified in the unique conditions of stormwater biofilters. This leaves the long-term efficiency of biofilter nitrogen treatment open to question and constrains the potential for future design improvements.

Biofiltration typically consists of a vegetated layer of sandy loam overlying sand and gravel layers, designed to capture, infiltrate and treat urban stormwater runoff before discharge downstream or into the surrounding environment or collection for harvesting [1], [2]. Like wastewater treatment wetlands, biofilters are engineered systems which harness natural biogeochemical processes. However, biofilters differ fundamentally from wetlands as a result of stormwater inflows and infiltration. While biofilters share some common design features with vertical flow wetlands, they are distinguished by being ephemeral, fed by urban intermittent stormwater runoff, which differs substantially from wastewater in composition and inflow hydrology [3], [4]. This leads to large, irregular variances in inundation, soil moisture and potentially nutrient, carbon and oxygen availability. As a result, biofilters are typically vegetated with terrestrial and semi-terrestrial plant species. Such differences likely alter the dominant nitrogen processes and drivers between treatment wetlands and stormwater biofilters. Characterising pollutant fate within stormwater biofilters is necessary not only for the optimal design of systems, but also to understand their long-term performance and determine suitable maintenance regimes.

Nitrogen is an essential nutrient in all biomass, but its natural cycling has been substantially altered by anthropogenic inputs and as a result forms a major contaminant of surface and ground waters [5]. Consequently, nitrogen processing has been extensively studied across terrestrial, semi-terrestrial and aquatic environments. This knowledge can be applied to infer possible nitrogen removal pathways in stormwater biofilters. Incoming nitrogen associated with urban stormwater runoff may undergo a range of potential fates, including assimilation, transformation by microbial processes (including nitrification, denitrification, dissimilatory nitrate reduction to ammonium (DNRA)), abiotic processes (including filtration and adsorption), or leaching from the system [6]. Based on research in other environments, the key fates for nitrate, a mobile inorganic form of nitrogen, are expected to be biotic assimilation (uptake by plants, bacteria, fungi or other microbes) and denitrification (conversion into gaseous forms primarily N_2_ or N_2_O) [7]. Assimilated nitrate is subsequently converted into an array of organic compounds and stored for some period, before return upon cell death or exudation. Decomposition processes act to either lock nitrogen up for longer term storage in recalcitrant components of the soil organic matter or re-release, when it is again available for uptake, transformation or leaching [6]. Hence, in many environments temporary storage from assimilation can occur over days, years, decades and beyond [6], [8]. However, in biofiltration systems concentrations of organic matter are initially very minimal in the engineered sandy substrate [9], and it is unknown if a pool will develop to provide significant long-term nitrogen storage. Other key nitrogen processes include nitrification and denitrification, which are both mediated by microbes, but require contrasting redox conditions. Many biofilter designs incorporate an upper drained layer underlain by a saturated layer, maintained using a raised outlet, which may theoretically provide zones for nitrification and denitrification respectively. A supplementary carbon source (e.g. wood chips, straw) is often mixed throughout the saturated zone to provide electrons for denitrification [10]. Despite increased nitrate removal associated with these design features [11], [12], to date no study has confirmed that this is due to denitrification. Without definitively relating design features to nitrogen processes, designers may be blind to opportunities for future performance enhancement. While biofilter design has progressed substantially over the past decade [9], [13], further improvements may be confined by the current lack of process knowledge. This is particularly the case for nitrogen, where the cyclical nature of assimilation and mineralisation – internal recycling – threatens to eventually overwhelm the demand for incoming nitrogen.

The removal performance quoted by most biofilter studies, which use a simple black-box input-output approach, is a reflection of predominantly short-term processes, ignoring possible long-term changes. While authors acknowledge the potential for re-mobilisation and leaching of previously attenuated nitrogen [14], [15], and several have investigated pollutant profiles [2], [16] or estimated plant accumulation [15], [17], none have explicitly characterised nitrogen fate to account for re-release. An initial step towards this understanding is determining the immediate fate of incoming nitrogen. While such an assessment focuses on rapid processing in a short timeframe, it has implications on longer term dynamics, given that a system cannot indefinitely accumulate nitrogen [18].

Assimilation and denitrification have been quantified in a range of engineered and natural systems. Denitrification has been reported to dominate processing in wastewater treatment wetlands [7], [19], wetlands treating high nitrate groundwater [20] and riparian soils receiving agricultural runoff [21]. In contrast, other studies have noted, sometimes surprisingly, the key role played by plant uptake in environments including streams [22], peat bogs [23], flooded soils planted with wetland species [24] and grassed buffer strips [25]. Factors driving the division may include carbon and nitrogen availability, nitrogen speciation, sediment characteristics, plant species morphology and physiology, plant density and hydrological regime [26], [27]. The dominant pathways have not been identified in biofilters, yet these influential factors may differ substantially under the characteristics of stormwater biofilters.

Given the ‘black-box’ approach of most biofiltration research to date, this experiment was designed to provide an initial investigation into nitrogen processes. The experiment aimed to quantify the immediate nitrate transformation pathways in stormwater biofilters, focusing upon the initial division between assimilation and denitrification, with the specific objectives to investigate the:

1. Effect of TN influent concentration on nitrate removal pathways in biofilter mesocosms planted with *Carex appressa*
2. Effect of plant species on nitrate removal pathways within laboratory-scale biofilters

This study uniquely applied isotope tracing techniques to stormwater biofilters in order to quantify processes, an approach which, to the authors' knowledge, has not previously been reported. In addition, few studies in other fields have applied isotope tracer across plant species, despite considerable interaction between species and nitrogen cycling [28]. Quantification across a loading gradient provides a basis to understand how results might vary across systems and inform comparisons. Overall, the study aimed to indicate if nitrogen may accumulate within components of the biofilter over time, which has critical implications for system design, maintenance and life span.

## Methods

### Experimental overview

This study is made up of two components. The first tested the effect of influent concentration on nitrate removal pathways in mesocosms planted with *Carex appressa*, selected as a high performing species in biofilters [14], [29]. In the second, nitrogen pathways were tracked in biofilter columns planted with various species. Both experiments use a laboratory-scale approach to provide insight into biofilter processes under controlled conditions, and include non-vegetated controls.

**Figure 1:**
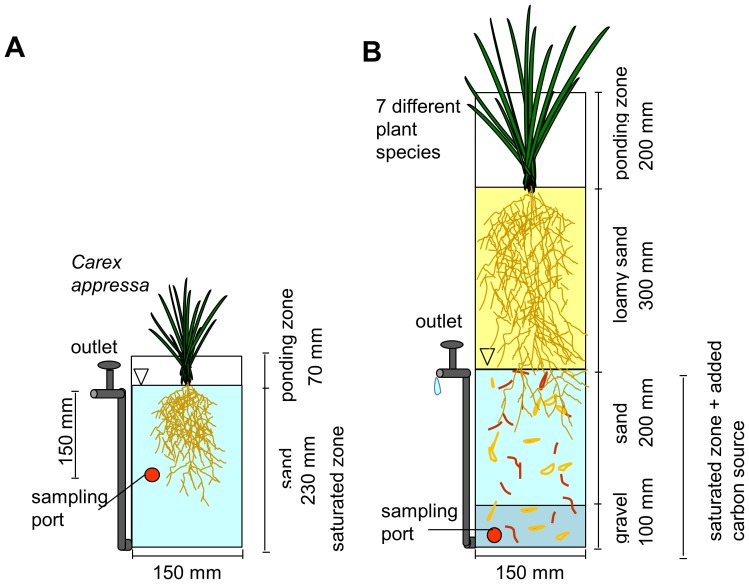
Experiment Configuration. A.) Influent concentration experiment (under fully saturated conditions) and B.) Multiple plant species experiment (with saturated zone overlaid by a non-saturated zone). Note diagrams are not drawn to scale.

Key differences between the experiments include their establishment period, depth and configuration of the saturated zone, replicate number, and the mixture of nitrogen species added (Table 1 and Figure 1). The *Carex appressa* influent concentration experiment was conducted under fully saturated conditions (Figure 1(a)), providing a simplified design to specifically investigate the effect of nitrate loading on biofilter saturated zone processes, which have been hypothesized as of primary importance in biofilter nitrogen removal performance [10], [12]. The experiment across plant species was established over a much longer period of plant growth and stormwater application, and incorporated an unsaturated layer above the saturated zone (typical of most vertical-flow stormwater biofilters, Figure 1 (b)).

**Table 1:** Summary of experiment details.

Experiment	1. Influent concentration	2. Multiple plant species
**Description**	Tested effect of 4 different influent N and P concentrations on NO_3_^−^ partitioning between denitrification, pore water and vegetation, using single plant species *Carex appressa* and non-vegetated controls.	Tested effect of 7 different plant species and non-vegetated control on NO_3_^−^ partitioning between denitrification, pore water and vegetation using constant influent composition of ‘typical’ stormwater.
	150 mm diameter PVC pipe containing 230 mm washed sand with constant saturation maintained.	150 mm diameter PVC pipe with layers of loamy sand, sand and gravel (Figure 1) across 600 mm. Saturated conditions maintained in lower 300 mm with sugar cane mulch and pine chips mixed throughout.
**Stormwater application**	Weekly dose of 1.63 L (∼one total pore volume)	Twice-weekly dose of 3.7 L (to Vic plant species, see below) and 4.2 L (to WA plant species) in accordance with local rainfall (∼one total pore volume)
	1 mg N/L, 0.3 mg P/L (non-vegetated control and ‘low’ dose)	∼2.2 mg N/L, 0.36 mg P/L (all columns)
	2 mg N/L, 0.6 mg P/L (‘medium’)	
	10 mg N/L, 2.8 mg P/L (‘high’)	
	20 mg N/L, 5.6 mg P/L (‘very high’)	
	Modified Long-Ashton nutrient solution [30] – (∼50% NO_3_^−^,50% NH_4_^+^ by weight)	Semi-natural urban stormwater with ‘typical’ components [3], [32] – (∼45% NO_3_^−^, 18% NH_4_^+^, 37% organic N by weight), ∼150 mg/L TSS
	Tracer added twice – July and August	Tracer added once - October
**Plant species**	*Carex appressa* (sedge)	*Carex appressa* (sedge; Vic)
		Palmetto Soft Leaf Buffalo (lawn grass/Vic)
		*Dianella revoluta* (sedge; Vic)
		*Juncus kraussii* (reed; WA)
		*Allocasurina littoralis* (tree; Vic)
		*Leptospermum continentale* (tree; Vic)
		*Hypocalymma angustifolium* (tree; WA)
**Initial plant growth period**	8 weeks	>17 months (includes 11 months in columns with twice-weekly of stormwater application)
**Location**	Controlled greenhouse	Open-air roofed greenhouse
**Number of replicates**	4 replicates	3 replicates

### Experimental setup

#### (i) Influent concentrations experiment

Twenty mesocosms (4 replicates for each tested nitrogen inflow concentration) were constructed using 150 mm diameter polyvinyl chloride (PVC) pipe and 230 mm of washed landscaping sand. Access holes were drilled and blocked using 12 mm butyl septa to allow sampling. A constant height of water was maintained to saturate the entire sediment. Two week old *Carex appressa* seedlings were planted into 16 of the mesocosms and grown for 8 weeks in a greenhouse alongside 4 non-vegetated controls.

#### (ii) Multiple plant species experiment

Twenty-four single-plant biofilter columns (3 replicates for each of the 7 tested plant species and non-vegetated control) were grown in an open-air roofed greenhouse for a period of 11 months. The plants were originally sourced as tubestock and established in planter bags (300 mm by 150 mm) using tapwater for 6 months. The columns were constructed using PVC pipe with a clear Perspex ponding zone and filled with loamy sand overlying sand and gravel layers. A raised outlet tap allowed a 300 mm saturated zone with a carbon source of pine chips and sugar cane mulch mixed throughout to form 5% by volume (Figure 1), typical of common stormwater biofilter design guidelines [9]. Seven plant species (Table 1) were selected to cover a range of plant forms and include both high and poor performing species for nitrogen removal in biofilters, determined from previous sampling (not presented here). These columns formed part of a broader 18 month study, which investigated nitrogen cycling across plant species and design configurations.

### Dosing and sampling

#### (i) Influent concentrations experiment

The mesocosms were dosed with varying concentrations of the modified Long-Ashton nutrient solution containing a 1∶1 ratio of Na^14^NO_3_∶Na^15^NO_3_ (Na^15^NO_3_>98% isotopic purity, Cambridge Isotope Laboratories), as described in Cavagnaro et al. [30]. The molar amount of NO_3_^−^ was matched with NH_4_^+^. Four vegetated mesocosms and four non-vegetated control mesocosms received the equivalent of 1 mg N/L (NO_3_^−^ and NH_4_^+^) and 0.3 mg P/L. This comprised the ‘Low’ inflow concentration. The remaining vegetated mesocosms were dosed with ‘Medium’ = 2 mg N/L, 0.6 mg P/L, ‘High’ = 10 mg N/L, 2.8 mg P/L and ‘Very high’ = 20 mg N/L, 5.6 mg P/L concentrations. This range of nutrient concentrations covers the typical range of urban stormwater runoff (1–3 mg N/L) [3] and beyond, towards concentrations more typical of wastewater. A weekly dose of 1.63 L was applied, which flushed the mesocosm by one pore volume based on a sand porosity of 0.4.

Before tracer addition, samples were collected from each inflow solution and the pore water, and analysed for background concentrations of NH_4_^+^, NO_3_^−^, N_2_ and N_2_O. Pore water samples were removed using a 25 mL plastic syringe with an 18-gauge needle. Samples for NH_4_^+^ and NO_3_^−^ were filtered through a 0.45 µm membrane filter (MicroAnalytix 30PS045AN) into a 12 mL container and frozen for subsequent analysis. Water samples for analysis of N_2_ and N_2_O concentrations were transferred into a 12.5 mL glass gas-tight vial (Exetainer, Labco). Zinc chloride (250 µL, 50% w/v) was added as a preservative [31]. This sampling regime was repeated 6 hourly for a period of 30 hours. Two pulses of isotope tracer were added, one in July and another in August 2012.

#### (ii) Multiple species experiment

The columns were dosed with semi-natural stormwater twice weekly, following the method outlined in Bratieres et al. [14] with a target Total Suspended Solids concentration of 150 mg/L and nutrient concentrations based on typical stormwater composition [3], [32]. The average nitrogen composition delivered is shown in Table 1 and comprised approximately 2.2 mg N/L and 0.36 mg P/L. The dose volume reflected a biofilter sized to 2.5% of its impervious catchment area, a twice weekly watering frequency and the annual average effective rainfall for Melbourne (Victoria) and Perth (Western Australia). Columns with Victorian (Vic) species were dosed with 3.7 L and Western Australian (WA) species with 4.2 L (Table 1). Buffalo, a common lawn grass, was cut regularly using scissors to simulate mowing. Following 11 months of stormwater dosing, the influent was enriched with Na^15^NO_3_^−^ in October 2011. Inflow samples were collected before and after tracer addition. Pore water samples were collected approximately 1.5 cm from the base of the saturated zone and processed as described for the previous experiment. An O_2_ minisensor (2.5 mm tip diameter) connected to a FireSting O_2_ oxygen meter (PyroScience GmbH, Germany) immediately measured dissolved oxygen concentrations in the sample. This sampling procedure was validated in the laboratory by collecting samples from anoxic water (created by 20 minutes of Argon gas bubbling). The anoxic water recorded an average of 1.4% air saturation (±0.1 standard deviation), but following sample collection using a syringe, plastic tubing and exetainer, the average dissolved oxygen concentration was 5.4% air saturation (±0.4), indicating the sampling procedure introduced a small amount of O_2_. Therefore, samples recording around 7% air saturation or lower can be considered anoxic. Sampling using a continuous oxygen probe was attempted but the fragile probes were repeatedly damaged when inserted into the biofilters. It should also be noted measurement of pH was not deemed necessary as stormwater influent and effluent from similar laboratory biofilters has been previously measured near neutral [33], CO_2_ production acts as a buffer against potentially low pH and denitrifiers operate under a wide range of conditions [34].

Six sets of samples were collected across 5 days from 25^th^–29^th^ October 2011. Samples were collected initially as each column finished draining, the next day in the morning and afternoon, then daily in the afternoon and following the next stormwater dosing (4 days after tracer addition).

The effluent from each column was sampled on 2^nd^ November 2011 to determine concentrations of total nitrogen (TN), total phosphorus (TP), filterable reactive phosphorus (FRP), total dissolved nitrogen (TDN), NH_4_^+^ and NO_x_, as per the method outlined in Payne et al. [26].

### Laboratory analyses - NH_4_^+^, NO_3_^−^, N_2_ and N_2_O

Concentrations of NH_4_^+^ and NO_x_ in the pore water were analysed using standard flow injection analysis methods in a NATA (National Association of Testing Authorities) accredited lab. Dissolved N_2_O, ^28^N_2_, ^29^N_2_ and ^30^N_2_ were quantified in the water samples after a 4 mL He (99.9%) headspace was placed in the vials and equilibrated by vigorously shaking for 5 minutes. The concentration of N_2_O was analysed by injecting a 100 µL sample of the headspace by gas chromatography (Hewlett Packard 5710A Gas Chromatograph). The total amount of N_2_O in the vials was calculated using Henry's law [35]. N_2_ was analysed on an ANCA GSL2 elemental analyser coupled to a Hydra 20–22 isotope ratio mass-spectrometer (IRMS; Sercon Ltd., UK).

### Data analysis

#### ^28^N_2_, ^29^N_2_ and ^30^N_2_ production and denitrification rates

A linear regression was fitted to the amount of excess ^29^N_2_ and ^30^N_2_ over time to calculate a production rate. The rate was adjusted to compensate for the loss rate, determined from a linear regression across the decline in labelled N_2_ in the later portion of the time series. The rate of ^15^N denitrification (D_15_), ^14^N denitrification (D_14_), the proportion of denitrification coupled to nitrification and total denitrification (D_total_) were calculated from the rates of ^30^N_2_ and ^29^N_2_ production, p ^30^N_2_ and p ^29^N_2_, respectively and the ratio of ^14^NO_3_^−^/^15^NO_3_^−^ [31].

#### Mass balance

**Figure 2:**
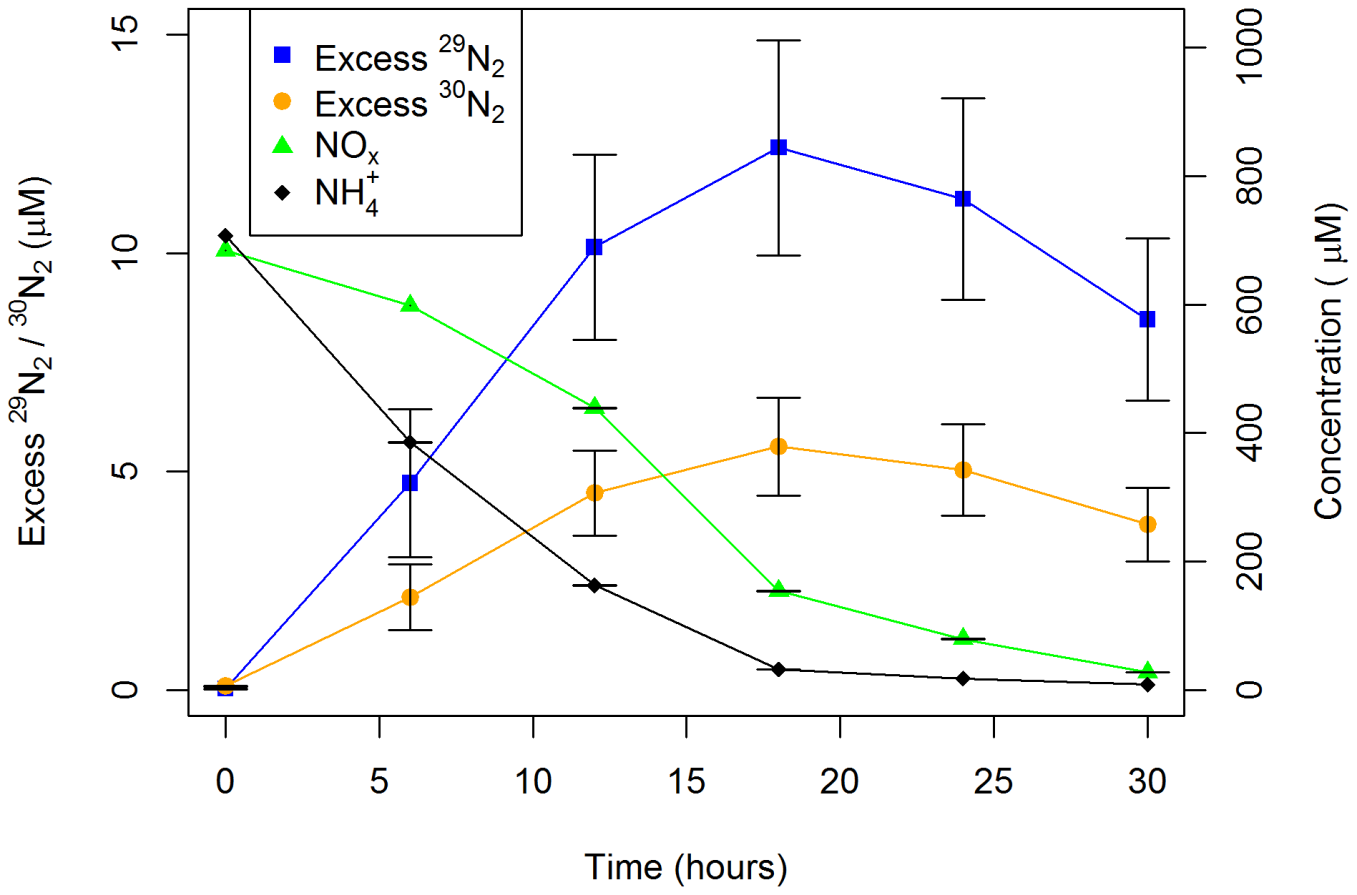
Nitrogen species concentrations. Examples of time series NH_4_^+^, NO_x_, excess ^29^N_2_ and ^30^N_2_ concentrations (± standard error (n = 4)) following dosing in the influent concentration experiment under very high nutrient dosing (20 mg N/L) measured in July 2012.

The mass balance was calculated over a period of 12 hours for all treatments, consistent with the period in which denitrification occurred in the *Carex appressa* mesocosm study (illustrated later by the rapid rise and peak in ^30^N_2_ and ^29^N_2_ in Figure 2). The total amount of ^15^N denitrified was calculated by integrating the rate of denitrification. This estimate of denitrification is expected to be conservative (i.e. an overestimate) given denitrification does not commence until anaerobic conditions establish, but achieved our objective of determining the maximum denitrification occurring within the systems. Both experiments were dosed with approximately one pore volume, resulting in negligible loss of ^15^N as outflow. The amount of ^15^NO_3_^−^ remaining in the pore water was calculated using the input ratio ^15^N∶^14+15^N and the pore water NO_3_^−^ concentration at 12 hours. If no sample was collected at this point, a concentration was linearly interpolated across the surrounding sampling time points. The proportion of ^15^NO_3_^−^ assimilated was calculated as the difference between the total amount of ^15^NO_3_^−^ added and the amount denitrified and remaining pore water ^15^NO_3_^−^.

#### Correlation and Significance Analysis

Pearson's product-moment correlation was used to determine if there was a relationship between the proportions assimilated and denitrified, and the proportions denitrified and remaining in the pore water. As the data were non-normally distributed in some cases, the use of Spearman rank correlation was used to confirm these. We tested for significant differences in assimilation between species, and in denitrification rate between nitrogen loadings using the non-parametric Kruskal-Wallis test. A critical value of α = 0.05 was used for hypothesis tests. Analyses were performed using the car Package [36] within the R Software Environment [37]. Michaelis-Menten curves were fitted using the drc Package [38] in R.

## Results

### Effect of TN influent concentration on nitrate removal pathways in *Carex appressa* mesocosms

Concentrations of NH_4_^+^ and NO_3_^−^ in the pore water decreased rapidly within 24 hours in all treatments, except the non-vegetated controls which produced NO_3_^−^, as a result of significant nitrification. Consistent with the decline in NO_3_^−^ in all vegetated treatments, ^29^N_2_ and ^30^N_2_ were produced over the first 12 hours after dosing, followed by a decrease in their concentrations thereafter (Figure 2 demonstrates these patterns for the very high nutrient treatment). Coupled nitrification-denitrification comprised approximately one third of denitrification in the high treatment. The production of N_2_O in the pore water represented an insignificant fraction of the nitrogen budget (<1%) and was therefore not considered further.

**Figure 3:**
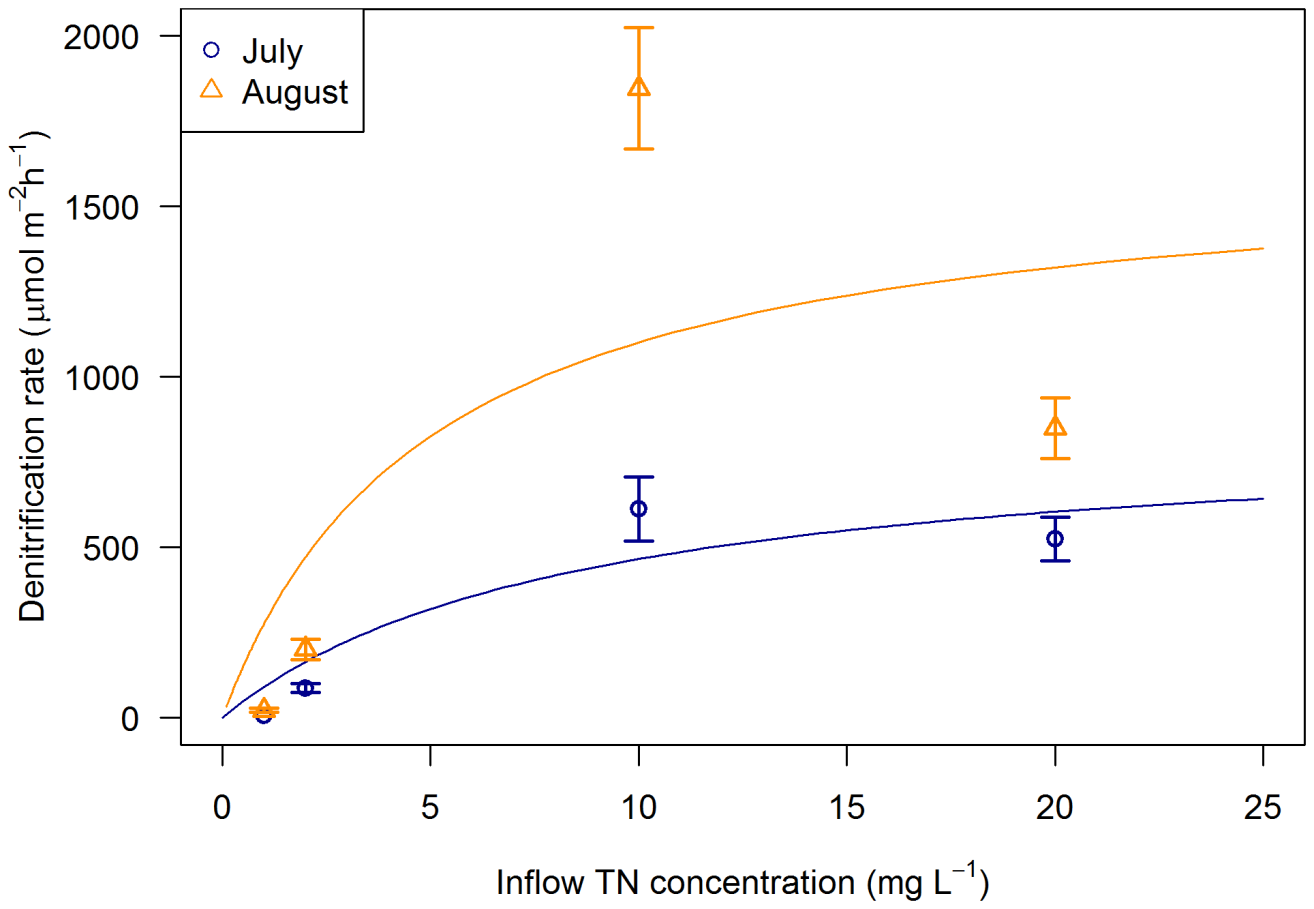
Rates of denitrification (^14^N+^15^N) against inflow TN concentration. Measured in the influent concentration experiment (± standard error (n = 4)) during July and August. Michaelis-Menten curves were fitted to give V_max_ = 861 µmol m^−2^ h^−1^ and K_m_ = 8.46 mg L^−1^ in July and V_max_ = 1653 µmol m^−2^ h^−1^ and K_m_ = 5.01 mg L^−1^ in August.

Rates of denitrification were extremely low in the control and low nutrient dosed columns (<25 µmol m^−2^ h^−1^), but increased sharply with higher nutrient loading, reaching a maximum rate in the high treatments (600 and 1800 µmol m^−2^ h^−1^, during July and August respectively) before decreasing in the very high treatment (Figure 3). There were significant differences in denitrification rate between the treatments in both July and August (p<0.05).

**Figure 4:**
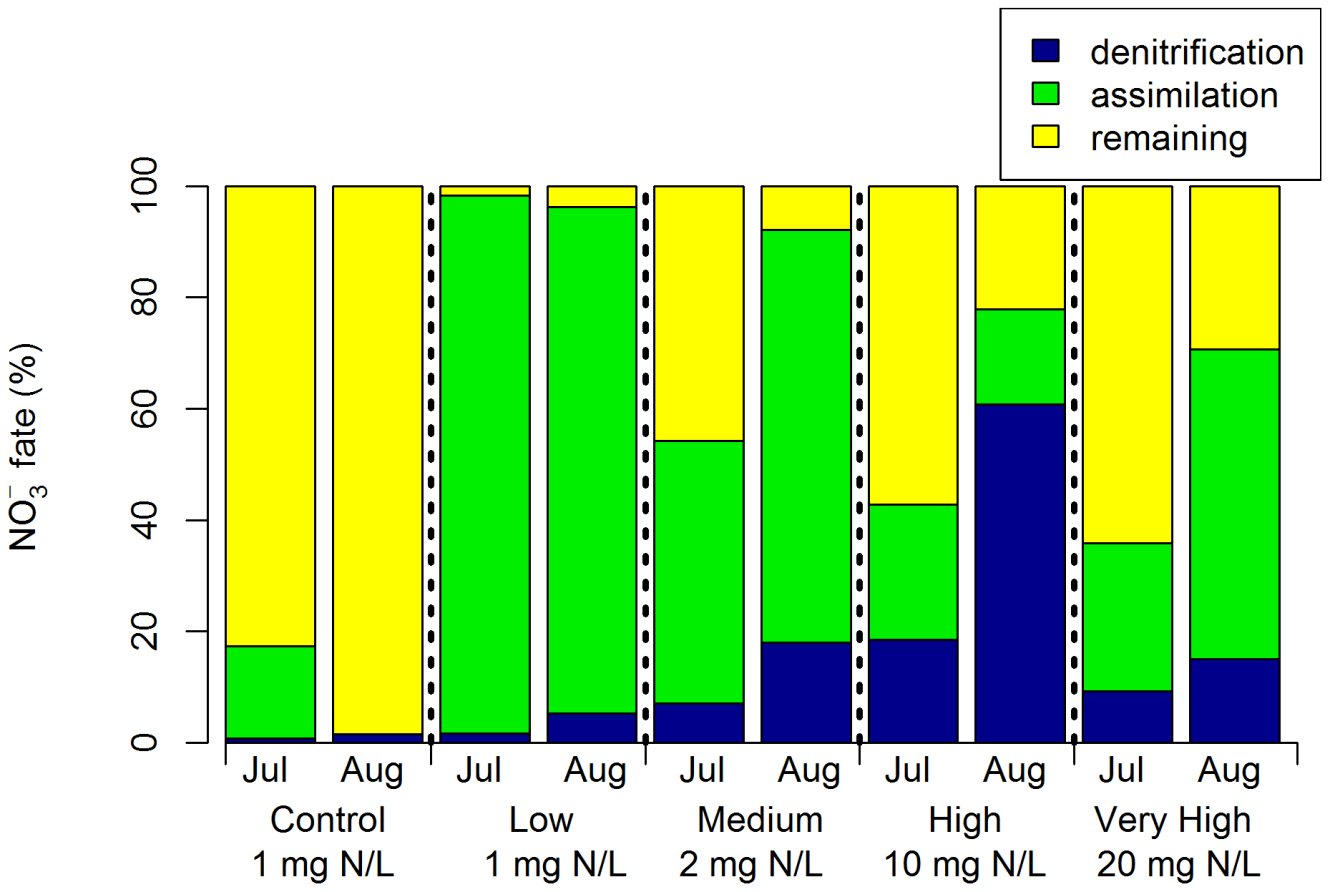
Division of ^15^NO_3_^−^ between denitrification, plant or microbial assimilation and remaining as ^15^NO_3_^−^ within the pore water. Measured 12(non-vegetated) and low, medium, high and very high (vegetated) nutrient dosing rates (n = 4).

There was negligible assimilation or denitrification of nitrate in the non-vegetated control columns; most nitrogen was recovered as NO_3_^−^ after 12 hours (Figure 4). In the vegetated columns, assimilation dominated in the low dose mesocosms, but generally decreased with higher loading. The fraction of ^15^NO_3_^−^ denitrified increased with nitrogen loading alongside the proportion remaining as nitrate. However, in the very high treatment denitrification declined and assimilation increased again.

### Effect of plant species on nitrate removal pathways within biofilters

The vegetated biofilter columns effectively reduced concentrations of TN and TP in the stormwater from 2.1 mg N/L and 0.31 mg P/L to averages of 0.27 mg N/L and 0.01 mg P/L respectively. NH_4_^+^ concentration reductions were high irrespective of plant species or the presence of vegetation, reduced from 0.4 mg N/L to <0.05 mg N/L. NO_x_ removal was also high but more variable; effluent concentrations ranged from 0.001–0.27 mg N/L from an influent concentration of 1.0 mg N/L. Removal of organic nitrogen was also effective with dissolved and particulate forms on average reduced from 0.4 mg N/L and 0.3 mg N/L to 0.12 mg N/L and 0.05 mg N/L respectively. The non-vegetated controls were less effective, with outflow concentrations averaging 1.47 mg N/L TN and a net production of NO_x_ to 1.27 mg N/L.

**Figure 5:**
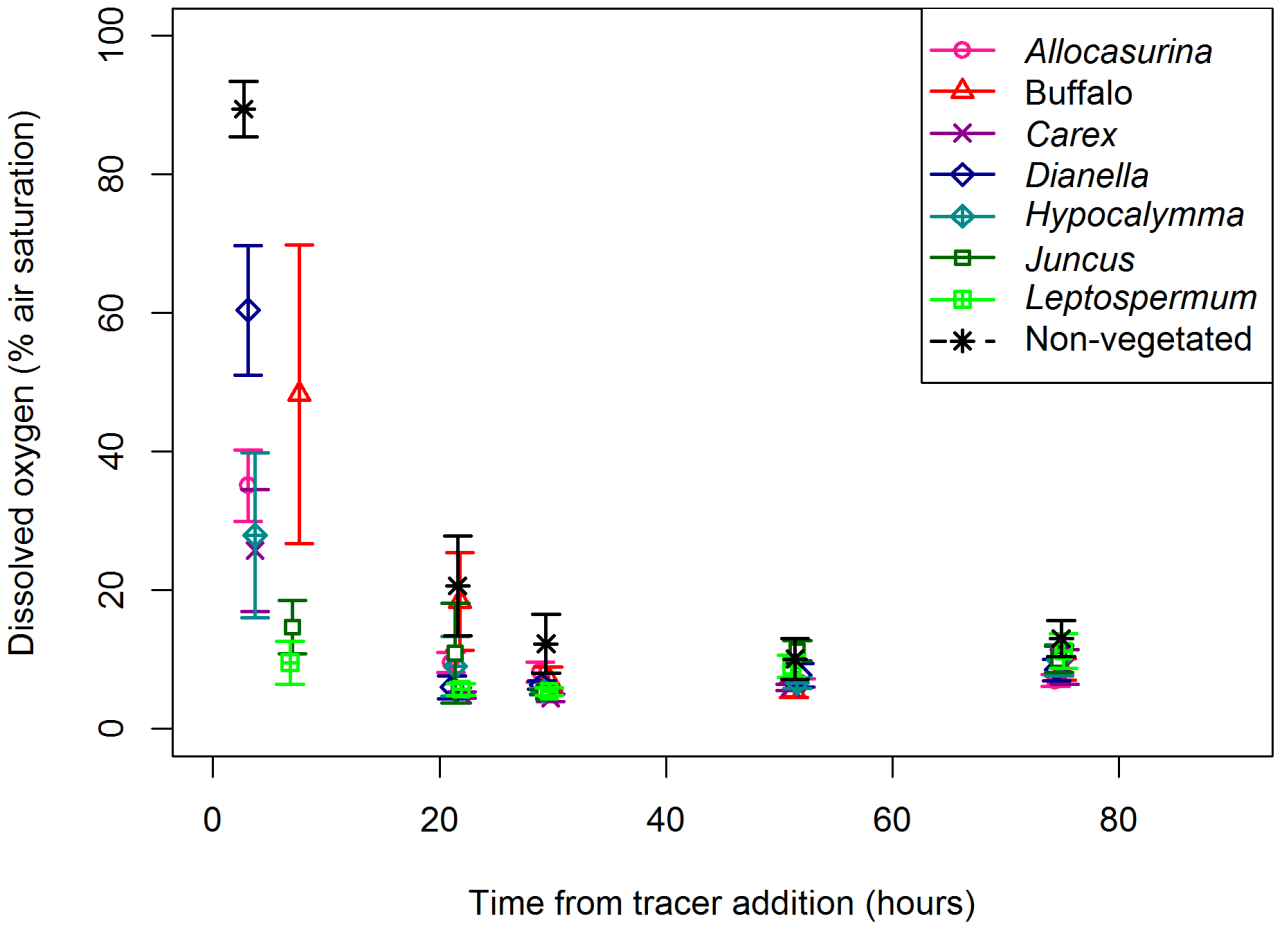
Change in pore water dissolved oxygen. Dissolved oxygen (% air saturated) (± standard error (n = 3) at base of columns across sampling period. Note the sample collection method introduced up to ∼7% air saturation.

Similar patterns for NO_3_^−^, ^29^N_2_ and ^30^N_2_ illustrated by Figure 2 for the pore water were also evident for the multiple species column experiment. Concentrations of NO_3_^−^ declined rapidly within 24 hours of tracer addition in all vegetated treatments, but remained elevated with some production in the non-vegetated controls, again indicating nitrification in these systems similarly to the mesocosm experiment. Concentrations of ^29^N_2_ and ^30^N_2_ increased rapidly, generally peaking between 25 and 45 hours, but continued to increase in the non-vegetated controls. N_2_O production was again minimal (<1% of ^15^NO_3_^−^) and was thus ignored in the mass balance. Concentrations of dissolved oxygen within the saturated zone rapidly declined towards anoxia, with a sharp decline measured across the first 22 hours for all species excluding Buffalo grass and the non-vegetated control which demonstrated a slower decline (Figure 5). Given the introduction of a small amount of oxygen during sample collection (up to ∼7% air saturation) and the expected commencement of denitrification <0.5 mg/L (or approximately <5% air saturation) [39], appropriate conditions for denitrification were considered to have occurred for most columns within 22 hours. In addition, the concentrations of dissolved oxygen in vegetated columns were significantly lower than in the non-vegetated columns across the first and second sampling times (collected an average of 5 hours and 22 hours after tracer addition respectively).

**Figure 6:**
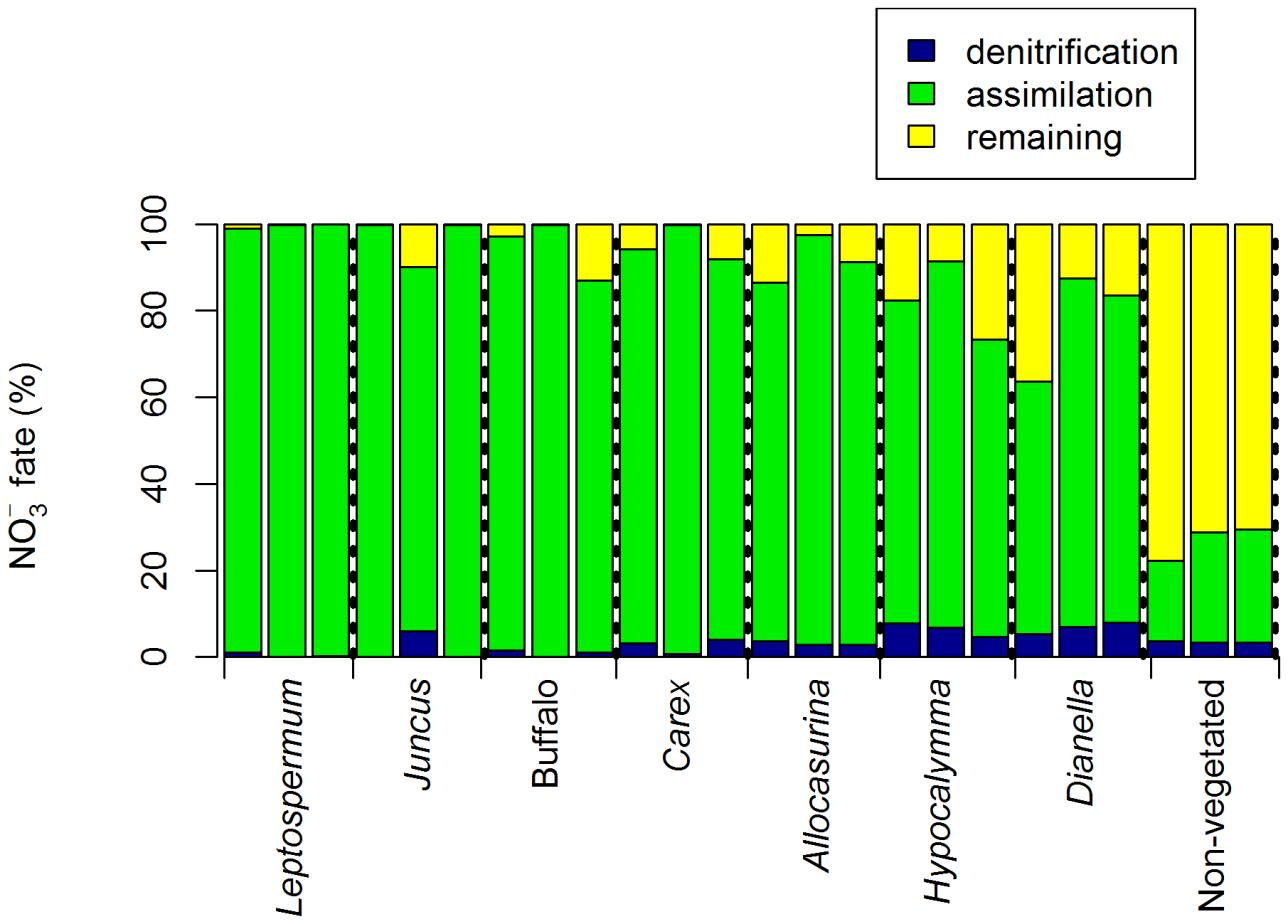
Division of ^15^NO_3_^−^ between denitrification, plant or microbial assimilation or remaining in the pore water. Results for each biofilter column in the multiple species experiment (3 replicates per species, n = 1).

Biotic assimilation was the primary fate of ^15^NO_3_^−^ in all vegetated columns; ranging between 58% and 100% of ^15^NO_3_^−^ (Figure 6). While an average of 88% (±7% standard error, n = 3) of ^15^NO_3_^−^ was assimilated, individual species differed significantly (p<0.05). The lowest uptake was associated with columns planted with *Dianella* and *Hypocalymma* which assimilated 58–80% and 69–85% respectively.

Assimilation and denitrification were inversely related (Supplementary information, Figure S1, r = −0.79, p<0.05). Denitrification was only a minor removal mechanism in the vegetated columns, providing a sink for, on average, only 3% (±2%) of ^15^NO_3_^−^ and ranging to a maximum of 5 to 8% across columns planted with *Dianella* and *Hypocalymma* species. These same treatments also had a greater proportion of ^15^NO_3_^−^ remaining in the pore water, demonstrated by a positive relationship between denitrification and nitrate remaining for vegetated columns (Figure S2, r = 0.66, p<0.05). In the non-vegetated controls assimilation was low, accounting for 19 to 26% of ^15^NO_3_^−^ fate. Instead ^15^NO_3_^−^ primarily remained in the pore water, and 3 to 4% was denitrified.

## Discussion

### Assimilation as a key biofilter pathway

Denitrification only formed a minor removal mechanism at typical stormwater concentrations in the biofilter columns and mesocosms. Biotic assimilation (uptake by plants and microbes) functioned as the major sink for incoming nitrate. The minimal contribution from denitrification is somewhat unexpected, given the focus on designing biofilters to promote denitrification [10], [12] and the dominance of denitrification in many treatment wetlands and some aquatic systems [7], [40]. Denitrification has been reported to account for 60–95% of removal in wastewater treatment wetlands [19], 89–96% in wetlands treating high nitrate groundwater [20], and up to 61–63% in riparian wetland soils treating agricultural runoff [21]. Denitrification can also be a critical process in semi-aquatic and terrestrial systems, including the soils of urban retention basins and parks [41].

However, the critical role of assimilation has also been noted in many studies, where plant and microbial assimilation make a significant, if not dominant, contribution to the removal of nitrate. This has been observed across riparian zones [42], flooded soils planted with wetland plants [24] and vegetated streams [22]. Assimilation accounted for 75% of nitrate retention in headwater streams [22], almost all nitrate deposited atmospherically on peat bogs [23] and was the primary removal mechanism in grassed buffers treating agricultural irrigation runoff [25].

The variation in nitrate fate between wastewater treatment wetlands and biofilters may result from their fundamental differences. While both are high nutrient, engineered and vegetated systems, biofilters generally experience greater moisture fluctuations, including prolonged drying. Hence, even with an underlying saturated zone, biofilter redox potential is dynamic. For optimal hydraulic and nutrient performance biofilter media is designed to be relatively free-draining with low organic matter or clay content [2]. As a result the system characteristics differ greatly from organic-rich and anoxic wetland sediments. Vertical sub-surface flow wetlands (which operate on similar principles to biofilters) can show particularly low denitrification relative to other treatment wetlands due to oxygenated conditions in the sediment [7], and this may similarly limit denitrification in the saturated zone of biofilters. While anoxic conditions develop (Figure 5), the influent is oxygenated.

It is questionable if assimilation will remain the dominant pathway throughout the biofilter lifespan. Denitrification may increase as organic matter accumulates [41], [43] and the uptake capacity of the plant biomass may diminish over time [27], [28]. The division between assimilation and denitrification will depend upon the magnitude of nitrogen immobilised in organic material and the availability of carbon, oxygen and nitrate. These dynamics may be sensitive to plant species and nitrate loading, as discussed further in the following sections.

In contrast to nitrate, ammonia reduction from the stormwater was effective regardless of the presence of vegetation or plant species. Removal processes included uptake, nitrification, and there was evidence of some coupled nitrification-denitrification in vegetated treatments.

### Adaptability of denitrifying bacteria

**Figure 7:**
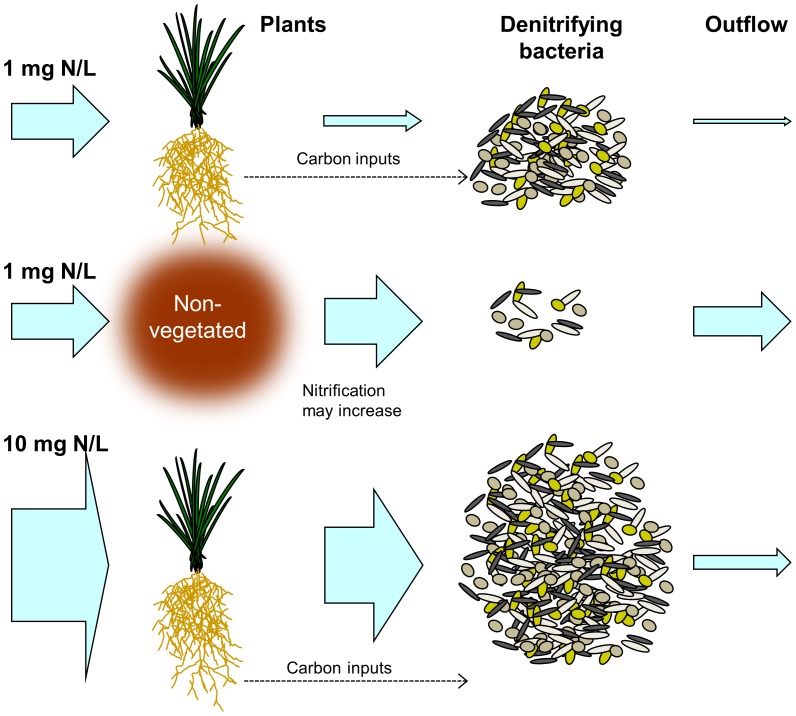
Conceptual diagram illustrating nitrate processing. Removal by assimilation and denitrification at different nitrogen loadings in vegetated and non-vegetated systems. The dependence of denitrifying bacteria on plant-derived carbon is also represented.

The importance of denitrification as a removal mechanism increased as nitrogen concentrations rose towards those of wastewater (∼10 mg N/L). The results suggest that the denitrifying bacteria are largely utilising the nitrate remaining after assimilation and that this portion grows as loading increases (Figure 7). In contrast, plant assimilation diminished in proportion – likely becoming constrained by other growth requirements or uptake rate after a critical point [7], [44]. Hence, denitrifying bacteria appear to have the adaptability to increase nitrate processing to a greater extent than plant and microbial assimilation, but remain dependent upon plant-derived carbon. As nitrate loading increases, the key role of plants may shift from assimilation to facilitation of denitrification.

The *Carex apressa* mesocosms represented a simplified experiment designed to investigate the influence of loading rate on the biofilter saturated zone; this experiment lacked the upper unsaturated layer or longer term loading of the multiple species biofilter column experiment, However, the consistency between the two experiments in terms of the proportion denitrified (7–18% mesocosms and 0–8% vegetated biofilter columns) and denitrification rate (88–202 µmol m^−2^ hr^−1^ and <1–635 µmol m^−2^ hr^−1^ respectively), when the same nitrate concentration was applied, suggests the results across the loading gradient can be meaningfully applied to biofiltration systems.

Other studies have also noted increases in denitrification across a loading gradient, but findings differ in the nature of this increase and relative change in assimilation. Denitrification and assimilation may both increase with loading, either linearly [22], or assimilation may increase at a much lower rate than denitrification [43], or denitrification rates may plateau (e.g. at loadings of around 5 mg N/L) [40], [44]. In the current study the denitrification rate appeared to increase rapidly, but reach a plateau, before declining at the highest loading rate (20 mg N/L comprising 10 mg NO_3_^−^-N/L). This may be due to a lower investment in root biomass by plants under the nutrient-rich conditions, which may result in less carbon to drive denitrification. Alternatively, the population of denitrifying bacteria may be inhibited by their generally facultative nature or other factors such as plant allelopathy, preventing optimal functioning in response to higher nitrate concentrations. However, data from the current study were inconclusive and testing these hypotheses will require further research. Nonetheless, the findings of the current study and others generally suggest that high loading leads to increased processing, although the proportion of nitrate retained by assimilation and denitrification is likely to decline across the loading spectrum as efficiency decreases [20], pathways saturate and mineralisation rates increase [44].

### Synergistic relationships

While the discussion has so far focused on the division between biotic assimilation and denitrification, the two processes are not independent. Plants can facilitate denitrification, either directly or coupled to nitrification, by carbon provision (root exudates or sloughed cells) and promoting heterogeneity in redox potential; anoxic (driven by intense heterotrophic decomposition) and oxic (due to root oxygen release by some species in waterlogged conditions) [45], [46]. This facilitative role of plants is highlighted by the performance of the non-vegetated control columns. In these treatments net nitrate production was commonly observed, indicating nitrification, which is facilitated by higher nitrate availability in the absence of plant-derived carbon (fuelling heterotrophic respiration) and plant assimilation (the small portion attributed to assimilation (Figure 6) was likely associated with bacteria consuming high C∶N ratio organic matter from the carbon source). Despite the availability of nitrate penetrating the saturated zone and the provision of a carbon source in the non-vegetated treatment, denitrification still failed to dominate processing and was higher in columns planted with *Hypocalymma* or *Dianella*. In the influent concentration experiment, which lacked a carbon source, denitrification was negligible in the non-vegetated controls. This illustrates the importance of root-derived carbon, which acts as a continuous and dynamic source, in driving denitrification, despite the competition from plant and microbial nitrate assimilation [21], [24]. This facilitation highlights the contradiction in the close relationship between plants and microbes; it is both synergistic and competitive, but essential and inter-dependent. Root exudation also hastens the onset of anaerobic conditions [46], as observed by the significantly lower concentrations of dissolved oxygen in the vegetated biofilter columns relative to the non-vegetated controls. However, a comparison of species rates of oxygen consumption and denitrification (Figures 5 and 6 respectively) yields no clear relationship (e.g. *Leptospermum*, *Juncus* and *Carex* demonstrate relatively rapid decline in dissolved oxygen but minimal denitrification, while the reverse is apparent for *Dianella*), suggesting available carbon is not exerting primary control on denitrification.

Do differences in kinetics or opportunity dictate the division between assimilation and denitrification? In the multiple species column experiment (and similar to biofilters in the field [9]) plants had the first opportunity to access incoming nitrate in the surface unsaturated layer before it reached the underlying saturated zone with high denitrification potential. In peat bogs this mechanism allowed a 5–10 cm layer of sphagnum moss to assimilate virtually all atmospheric nitrate additions despite favourable conditions for denitrification in the underlying soil [23]. In addition, the stormwater influent oxygenated the saturated zone, delaying the onset of suitable anaerobic conditions for denitrification (Figure 5), which may have advantaged biotic assimilation irrespective of rates. Hence, the kinetic rates of assimilation and denitrification cannot be conclusively compared in the current study. Regardless, biotic assimilation of ammonium appeared more rapid than nitrate (Figure 2), as expected due to general plant preference for ammonium [24].

### Plant species variation

The proportion of nitrate assimilated displayed differences among plant species, from an average 99% uptake by *Leptospermum* to 74% for *Hypocalymma* and *Dianella* treatment replicates (Supplementary information, Figure S3). Interestingly, species demonstrating higher nitrate assimilation (83–99% ^15^NO_3_^−^ processing) also tended to be more effective for use in biofilters (i.e. less nitrate remaining in the pore water) (*Leptospermum, Carex*, Buffalo lawn grass, *Juncus* and *Allocasurina*), despite minimal denitrification (0–6%) (Figures S4 and S5). Alongside less effective species (*Dianella* and *Hypocalymma*) assimilation was reduced (58–85%), and while denitrification increased (5–8%), it did not compensate entirely, leading to increased nitrate concentrations in the pore water (9–36%). The minimal contribution of denitrification to performance was unexpected, but is compatible with the hypothesis that denitrifying bacteria primarily receive any nitrate left over after assimilation. Many characteristics may contribute to plant species variation including morphological (such as root architecture, biomass and diameter profile) and physiological traits (including root oxygenation, biomass composition, plant strategy, seasonality, assimilation rate, nitrogen preference, mycorrhizal symbiosis and photosynthate partitioning) [28], [45], [47]. Studies have already identified the importance of the root zone (rhizosphere) [48] or correlated biofilter efficiency for nitrogen with long, deep roots, a high root biomass and rapid growth [29], and further relationships are the subject of a current study.

### Organic nitrogen stores – beneficial or false economy?

Plant nitrogen uptake and release follow seasonal cycles and the effect in wetlands has been likened to a ‘spiral’ [49]. The process acts to attenuate nitrogen within the system [49] and convert inorganic forms to an array of organic compounds. The significant impact of these plant services are recognised across wetlands, aquatic systems and vegetated buffers [7], [22], [25]. These functions may be particularly beneficial in biofilters, given that inflow events are relatively intermittent and transient. Assimilation will slow nitrogen passage through the biofilter and re-release will occur over a relatively longer timescale, which may facilitate more effective microbial processing. The benefits of temporary nitrogen immobilisation in plants are also well acknowledged in agriculture through the use of cover crops, which are used to minimise nitrate leaching between main crops and increasingly applied to protect groundwater quality [8].

However, the benefits of storage in the biomass may extend well beyond short seasonal or annual cycles if incorporated into recalcitrant compounds. These may be stored over the long term in woody biomass [44] or incorporated into the soil organic matter (SOM), which may exceed the nitrogen storage capacity of the plant biomass by a factor of 10 [50]. Such stores could potentially endure beyond the biofilter lifespan (generally 15–20 years [9]). In treatment wetlands, the accumulation of organic material, alongside denitrification, can both form significant pathways for nitrogen removal over the long term, even under high loading [43]. However, due to low anaerobic decomposition rates, wetlands are natural sinks for organic material [6] – it is less certain if significant accumulation will occur in the ephemeral environment of biofilters.

On the other hand, the conversion and attenuation function of plants leads to the production of nitrogen forms that require multiple processing steps before permanent removal is possible via denitrification. In particular, loss of dissolved organic nitrogen from the system is a high risk unless efficient mineralisation occurs [25]. In addition, both plants and microbes may over time increasingly source nitrogen from internal cycling processes. Harvesting the plant biomass to remove nitrogen could extend plant nitrogen demand, as has been observed in grassed systems [51]. In biofilters, however, this may be constrained by cost, the likelihood of filter media compaction [2] and concerns over pollution translocation [52].

### What does this mean for long term biofilter function?

This experiment was limited by constraints inherent in studies at the laboratory scale, including small biofilter surface area, single-plant columns, regular inflows and the short time frame of the tracer experiment. In light of this, do the findings have any implications on processes in mature field-scale biofilters?

Concentrations of TN in the effluent from vegetated columns in the current study (0.11–0.45 mg N/L) are much lower than previous laboratory and field studies (typically 1 mg N/L at best [1], [53]). This difference may in part reflect a design change towards media with a high sand and low nutrient content, which minimises nutrient leaching [9], [26]. However, the current results do require validation under field conditions and extension by tracing nitrogen fate over longer periods. Nevertheless, the findings form an initial step in identifying and quantifying biofilter nitrogen processes, and thus represent an important advance on the predominantly “black-box” approach of studies to date.

The experiment quantified denitrification in the first hours following an inflow event. Within the multiple species column experiment the vegetated biofilters functioned effectively and little nitrate remained in the pore water after 24 hours (Figure 6), suggesting rapid initial processing, particularly alongside the most effective plant species. Given the transient nature of biofilter inflows, rapid initial processing may be an inherent characteristic of biofilters, possibly more so than it is for treatment wetlands. Hence, the results of the experiment may have some realistic implications on longer-term nitrogen fate in biofilters.

If denitrification does not form a significant long-term nitrogen sink in biofilters, ongoing performance is heavily dependent upon the capacity and duration of the biomass storage. Studies of ecosystem succession suggest both mature and early-stage systems have limited retentive capacity, and intermediate systems have the greatest potential to capture nitrogen [54]. The storage capacity increases over time as plants and a pool of soil organic matter establish [55], [56]. However, growth of these storages will eventually plateau as the system moves towards a steady state [54] and nitrogen saturation [18]. At this point nitrogen returns (e.g. net nitrification) counteract the net biotic uptake, such that system inputs again equal outputs (e.g. leaching) [18]. Sustained high nutrient loading will exacerbate the saturation process and impair long-term functionality [44].

It might be expected that biofilters will similarly display an increase in performance towards this intermediate state, followed by a gradual decline in performance. However, do biofilters follow these same successional patterns and if so, is the point of zero net retention reached within the biofilter lifespan? These succession theories were developed for terrestrial forests where denitrification is negligible. If extrapolated to ephemeral biofiltration systems, the peak in performance may be sustained over a longer period of time if denitrification increases to counteract the decline in net biotic uptake.

### Further work

The minor role of denitrification in early biofilter life lends greater urgency to the need to quantify nitrogen processing across the entire biofilter lifespan. If assimilation continues to be a major pathway, the temporal and quantitative dynamics of storage in the biomass or soil organic matter need to be delineated. The return flux of nitrogen from mineralisation should be incorporated into assessments of biofilter lifetime performance. In addition to the techniques employed in this paper, further understanding can also be gained using molecular biology techniques (such as qPCR), which are capable of characterising the bacterial community. As this study has demonstrated, plant species and loading can be critical influences on processes. Hence, the interconnectedness between assimilation, denitrification, plant species and cumulative loading over extended time periods require further research.

## Conclusions

This study is the first known to apply a nitrate isotope tracer to the quantification of internal stormwater biofilter processes. Nitrate processing varied significantly with plant species and influent nitrogen concentration. Denitrification was only responsible for processing 0–8% of incoming nitrate in the laboratory-scale stormwater biofilters, suggesting biotic assimilation is the primary sink. Species identified as effective for reducing effluent concentrations (e.g. *Leptospermum, Carex*, Buffalo, *Juncus, Allocasurina*) tended to be associated with higher nitrate assimilation and minimal denitrification. This is surprising, given past efforts in biofilter design to promote denitrification. Instead, the denitrifying bacteria in the underlying saturated zone of biofilters appear to receive only the nitrate remaining after assimilation, such that nitrate plays a more important role in biofilters planted with species shown to be less effective in nitrogen removal. Higher nitrate loads increased the relative contribution of denitrification, implying denitrifying bacteria have greater adaptability to process high concentrations, whereas biotic assimilation becomes overwhelmed. While the results contrast with wastewater treatment wetlands, where microbial processing commonly dominates under higher loading, they are compatible with other studies highlighting the importance of plant assimilation as a nitrogen conversion mechanism and either a temporary or long-term (in soil organic matter or woody biomass) storage. This distinction from wetland functioning may reflect the unique characteristics of biofilters as quasi-terrestrial, engineered, ephemeral and highly-dynamic systems. The results have significant implications for biofilter design, maintenance and lifespan. With biotic assimilation dominating processing in early biofilter life, the need to characterise long-term organic matter accumulation and decomposition, the influence of plant species and prevalence of denitrification in mature systems becomes far more critical.

## Supporting Information

Figure S1 **Relationship between percentage of ^15^NO_3_^−^ denitrified and assimilated.** Results for each biofilter column in the multiple species experiment (3 replicates per species, n = 1). Linear regression line fitted to the vegetated treatments only.
(TIFF)

Figure S2 **Relationship between percentage of ^15^NO_3_^−^ remaining in the porewater and denitrified.** Results for each biofilter column in the multiple species experiment (3 replicates per species, n = 1). Linear regression line fitted to the vegetated treatments only.
(TIFF)

Figure S3 **Boxplot comparison of percentage of ^15^NO_3_^−^ assimilated across the vegetated treatments.** Results for replicated columns in the multiple species experiment (3 replicates per species, n = 3).
(TIFF)

Figure S4 **Boxplot comparison of percentage of ^15^NO_3_^−^ denitrified across the vegetated treatments.** Results for replicated columns in the multiple species experiment (3 replicates per species, n = 3).
(TIFF)

Figure S5 **Boxplot comparison of percentage of ^15^NO_3_^−^ remaining in the porewater across the vegetated treatments.** Results for replicated columns in the multiple species experiment (3 replicates per species, n = 3).
(TIFF)

## References

[1] Hunt WF, Smith JT, Jadlocki SJ, Hathaway JM, Eubanks PR (2008) Pollutant Removal and Peak Flow Mitigation by a Bioretention Cell in Urban Charlotte, N.C. Journal of Environmental Engineering 134: 403–408.

[2] Hatt BE, Fletcher TD, Deletic A (2008) Hydraulic and pollutant removal performance of fine media stormwater filtration systems. Environmental Science and Technology 42: 2535–2541. 18504993 10.1021/es071264p

[3] Taylor GD, Fletcher TD, Wong THF, Breen PF, Duncan HP (2005) Nitrogen composition in urban runoff–implications for stormwater management. Water Research 39: 1982–1989. 15921721 10.1016/j.watres.2005.03.022

[4] Gray SR, Becker NSC (2002) Contaminant flows in urban residential water systems. Urban Water 4: 331–346.

[5] Vitousek PM, Aber JD, Howarth RW, Likens GE, Matson PA, et al (1997) Human alteration of the global nitrogen cycle: Sources and consequences. Ecological Applications 7: 737–750.

[6] Reddy KR, DeLaune RD (2008) Biogeochemistry of wetlands science and applications. Boca Raton: Taylor & Francis, 2008.

[7] Vymazal J (2007) Removal of nutrients in various types of constructed wetlands. Science of the Total Environment 380: 48–65. 17078997 10.1016/j.scitotenv.2006.09.014

[8] Powlson DS (1993) Understanding the soil nitrogen cycle. Soil Use and Management 9: 86–93.

[9] FAWB (2009) Adoption Guidelines for Stormwater Biofiltration Systems: Facility for Advancing Water Biofiltration, Monash University, June 2009.

[10] Kim H, Seagren EA, Davis AP (2003) Engineered bioretention for removal of nitrate from stormwater runoff. Water Environment Research 75: 355. 12934829 10.2175/106143003x141169

[11] Zinger T, Fletcher TD, Deletic A, Blecken GT, Viklander M (2007) Optimisation of the nitrogen retention capacity of stormwater biofiltration systems. Lyon, France.

[12] Zinger Y, Blecken GT, Fletcher TD, Viklander M, Deletic A (2013) Optimising nitrogen removal in existing stormwater biofilters: Benefits and tradeoffs of a retrofitted saturated zone. Ecological Engineering 51: 75–82.

[13] Davis AP, Hunt WF, Traver RG, Clar M (2009) Bioretention Technology: Overview of Current Practice and Future Needs. Journal of Environmental Engineering 135: 109–117.

[14] Bratières K, Fletcher TD, Deletic A, Zinger Y (2008) Nutrient and sediment removal by stormwater biofilters: A large-scale design optimisation study. Water Research 42: 3930–3940. 18710778 10.1016/j.watres.2008.06.009

[15] Davis AP, Shokouhian M, Sharma H, Minami C (2006) Water Quality Improvement through Bioretention Media: Nitrogen and Phosphorus Removal. Water Environment Research 78: 284. 16629269 10.2175/106143005x94376

[16] Davis AP, Shokouhian M, Sharma H, Minami C (2001) Laboratory study of biological retention for urban stormwater management. Water Environment Research 73: 5. 11558302 10.2175/106143001x138624

[17] Lucas WC, Greenway M (2011) Hydraulic Response and Nitrogen Retention in Bioretention Mesocosms with Regulated Outlets: Part I - Hydraulic Response. Water Environment Research 83: 692–702. 21905406

[18] Aber J, McDowell W, Nadelhoffer K, Alison M, Berntson G, et al (1998) Nitrogen Saturation in Temperate Forest Ecosystems. Bioscience 48: 921–934.

[19] Lee C-g, Fletcher TD, Sun G (2009) Nitrogen removal in constructed wetland systems. Engineering in Life Sciences 9: 11–22.

[20] Lin Y-F, Jing S-R, Wang T-W, Lee D-Y (2002) Effects of macrophytes and external carbon sources on nitrate removal from groundwater in constructed wetlands. Environmental Pollution 119: 413–420. 12166674 10.1016/s0269-7491(01)00299-8

[21] Matheson FE, Nguyen ML, Cooper AB, Burt TP, Bull DC (2002) Fate of ^15^N-nitrate in unplanted, planted and harvested riparian wetland soil microcosms. Ecological Engineering 19: 249–264.

[22] Cooper AB, Cooke JG (1984) Nitrate loss and transformation in 2 vegetated headwater streams. New Zealand Journal of Marine and Freshwater Research 18: 441–450.

[23] Urban NR, Eisenreich SJ, Bayley SE (1988) The Relative Importance of Denitrification and Nitrate Assimilation in Midcontinental Bogs. Limnology and Oceanography 33: 1611–1617.

[24] Kirk GJD, Kronzucker HJ (2005) The Potential for Nitrification and Nitrate Uptake in the Rhizosphere of Wetland Plants: A Modelling Study. Annals of Botany 96: 639–646. 16024557 10.1093/aob/mci216 PMC4247031

[25] Bedard-Haughn A, Tate KW, Kessel Cv (2004) Using Nitrogen-15 to Quantify Vegetative Buffer Effectiveness for Sequestering Nitrogen in Runoff. Journal of Environmental Quality 33: 2252–2262. 15537948 10.2134/jeq2004.2252

[26] Payne EGI, Pham T, Cook PLM, Fletcher TD, Hatt BE, et al (in press) Biofilter design for effective nitrogen removal from stormwater - influence of plant species, inflow hydrology and use of a saturated zone. Water Science and Technology 10.2166/wst.2014.013 24647199

[27] Mitsch WJ, Gosselink JG (2000) Wetlands. New York, United States of America: John Wiley & Sons, Inc.

[28] Payne EGI, Fletcher TD, Cook PLM, Deletic A, Hatt BE (in press) Processes and drivers of nitrogen removal in stormwater biofiltration. Critical Reviews in Environmental Science and Technology

[29] Read J, Fletcher TD, Wevill T, Deletic A (2010) Plant Traits that Enhance Pollutant Removal from Stormwater in Biofiltration Systems. International Journal of Phytoremediation 12: 34–53. 20734627 10.1080/15226510902767114

[30] Cavagnaro TR, Smith FA, Lorimer MF, Haskard KA, Ayling SM, et al (2001) Quantitative Development of *Paris*-Type Arbuscular Mycorrhizas Formed between *Asphodelus fistulosus* and *Glomus coronatum* . New Phytologist 149: 105–113. 10.1046/j.1469-8137.2001.00001.x 33853237

[31] Nielsen LP (1992) Denitrification in sediment determined from nitrogen isotope pairing. FEMS Microbiology Letters 86: 357–362.

[32] Duncan HP (1999) Urban Stormwater Quality: A Statistical Overview. Melbourne, Australia: Cooperative Research Centre for Catchment Hydrology.

[33] Blecken G-T, Zinger Y, Deletic A, Fletcher TD, Viklander M (2009) Impact of a submerged zone and a carbon source on heavy metal removal in stormwater biofilters. Ecological Engineering 35: 769–778.

[34] Šimek M, Jíšová L, Hopkins DW (2002) What is the so-called optimum pH for denitrification in soil? Soil Biology and Biochemistry 34: 1227–1234.

[35] Weiss RF, Price BA (1980) Nitrous oxide solubility in water and seawater. Marine Chemistry 8: 347–359.

[36] Fox J, Weisberg S (2011) An {R} Companion to Applied Regression. Thousand Oaks, CA: Sage.

[37] R Core Development Team (2012) R: A language and environment for statistical computing. Vienna, Austria: R Foundation for Stastistical Computing

[38] Ritz C, Streibig JC (2005) Bioassay Analysis using R. Journal of Statistical Software 12.

[39] Seitzinger SP (1988) Denitrification in Freshwater and Coastal Marine Ecosystems: Ecological and Geochemical Significance. Limnology and Oceanography 33: 702–724.

[40] Kreiling R, Richardson W, Cavanaugh J, Bartsch L (2011) Summer nitrate uptake and denitrification in an upper Mississippi River backwater lake: the role of rooted aquatic vegetation. Biogeochemistry 104: 309–324.

[41] Zhu W-X, Dillard N, Grimm N (2004) Urban nitrogen biogeochemistry: status and processes in green retention basins. Biogeochemistry 71: 177–196.

[42] Pinay G, Ruffinoni C, Wondzell S, Gazelle F (1998) Change in Groundwater Nitrate Concentration in a Large River Floodplain: Denitrification, Uptake, or Mixing? Journal of the North American Benthological Society 17: 179–189.

[43] Craft CB (1997) Dynamics of nitrogen and phosphorus retention during wetland ecosystem succession. Wetlands Ecology and Management 4: 177–187.

[44] Bernot M, Dodds W (2005) Nitrogen Retention, Removal, and Saturation in Lotic Ecosystems. Ecosystems 8: 442–453.

[45] Reddy KR, Patrick WH Jr, Lindau CW (1989) Nitrification-denitrification at the plant root-sediment interface in wetlands. Limnology & Oceanography 34: 1004–1013.

[46] Minett DA, Cook PL, Kessler AJ, Cavagnaro TR (2013) Root effects on the spatial and temporal dynamics of oxygen in sand-based laboratory-scale constructed biofilters. Ecological Engineering 58: 414–422.

[47] Tanner CC (1996) Plants for constructed wetland treatment systems — A comparison of the growth and nutrient uptake of eight emergent species. Ecological Engineering 7: 59–83.

[48] Lucas W, Greenway M (2008) Nutrient Retention in Vegetated and Nonvegetated Bioretention Mesocosms. Journal of Irrigation and Drainage Engineering 134: 613–623.

[49] Kadlec RH, Tanner CC, Hally VM, Gibbs MM (2005) Nitrogen spiraling in subsurface-flow constructed wetlands: Implications for treatment response. Ecological Engineering 25: 365–381.

[50] Jenkinson DS (1990) An introduction to the global nitrogen cycle. Soil Use and Management 6: 56–61.

[51] Bedard-Haughn A, Tate KW, Kessel Cv (2005) Quantifying the Impact of Regular Cutting on Vegetative Buffer Efficacy for Nitrogen-15 Sequestration. Journal of Environmental Quality 34: 1651–1664. 16091618 10.2134/jeq2005.0033

[52] Collins KA, Lawrence TJ, Stander EK, Jontos RJ, Kaushal SS, et al (2010) Opportunities and challenges for managing nitrogen in urban stormwater: A review and synthesis. Ecological Engineering 36: 1507–1519.

[53] Roberts SJ, Fletcher TD, Garnett L (2012) Bioretention saturated zones; do they work at the large-scale? 7th International Conference on Water Sensitive Urban Design 21–23 February 2012. Melbourne, Australia.

[54] Vitousek PM, Reiners WA (1975) Ecosystem Succession and Nutrient Retention: A Hypothesis. Bioscience 25: 376–381.

[55] Odum EP (1969) The strategy of ecosystem development. Science 164: 262–270. 5776636 10.1126/science.164.3877.262

[56] Mitsch WJ, Day JW, Zhang L, Lane RR (2005) Nitrate-nitrogen retention in wetlands in the Mississippi River Basin. Ecological Engineering 24: 267–278.

