# Supplementary figures and images for: Temporary Storage or Permanent Removal? The Division of Nitrogen between Biotic Assimilation and Denitrification in Stormwater Biofiltration Systems
Source: PLoS One. 2014 Mar 26;9(3):e90890. doi: 10.1371/journal.pone.0090890 (PMC3966729; doi:10.1371/journal.pone.0090890)

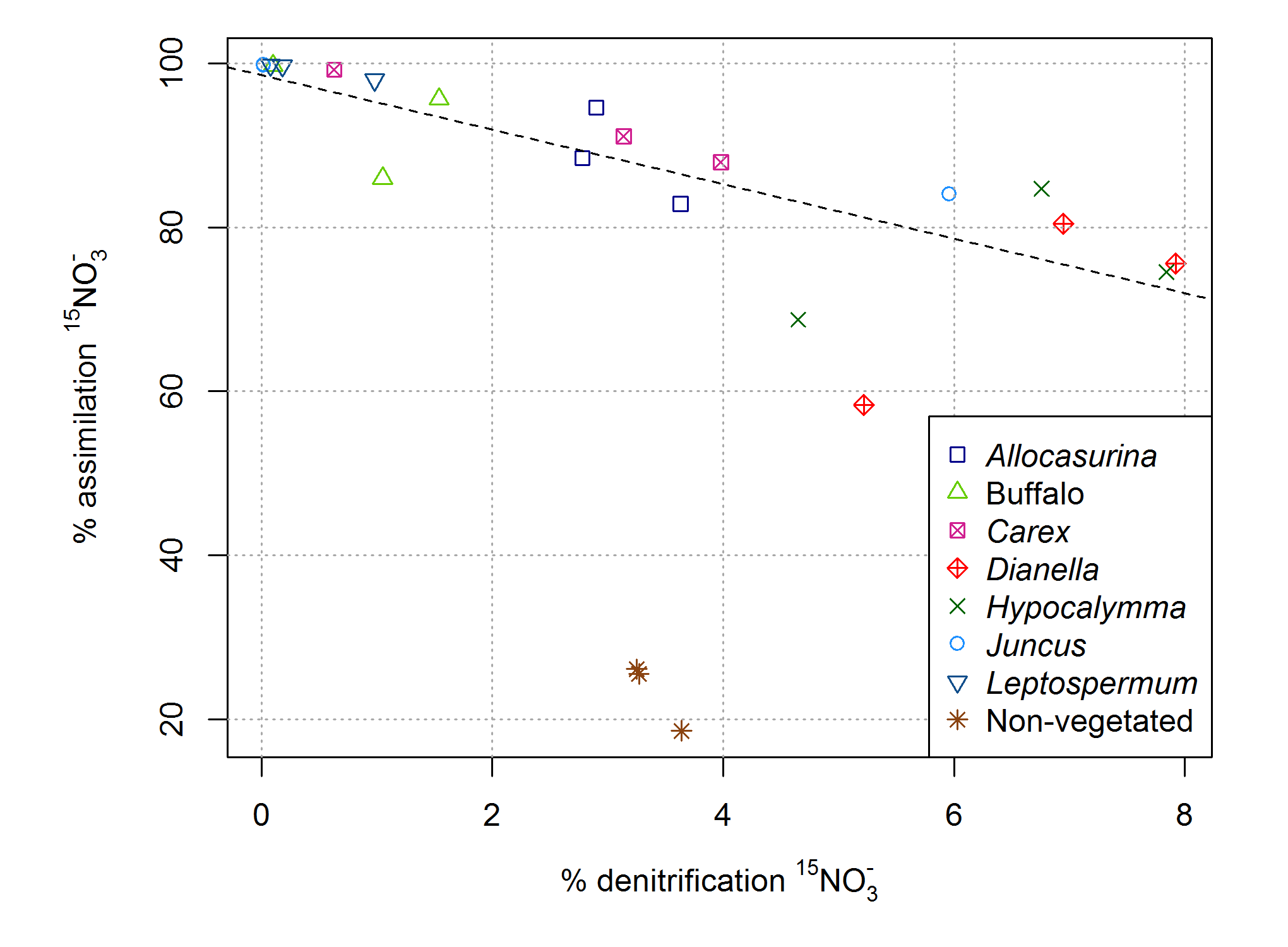

Supplement: Figure S1 — Relationship between percentage of 15NO3− denitrified and assimilated. Results for each biofilter column in the multiple species experiment (3 replicates per species, n = 1). Linear regression line fitted to the vegetated treatments only. (TIFF) [file pone.0090890.s001.tiff]

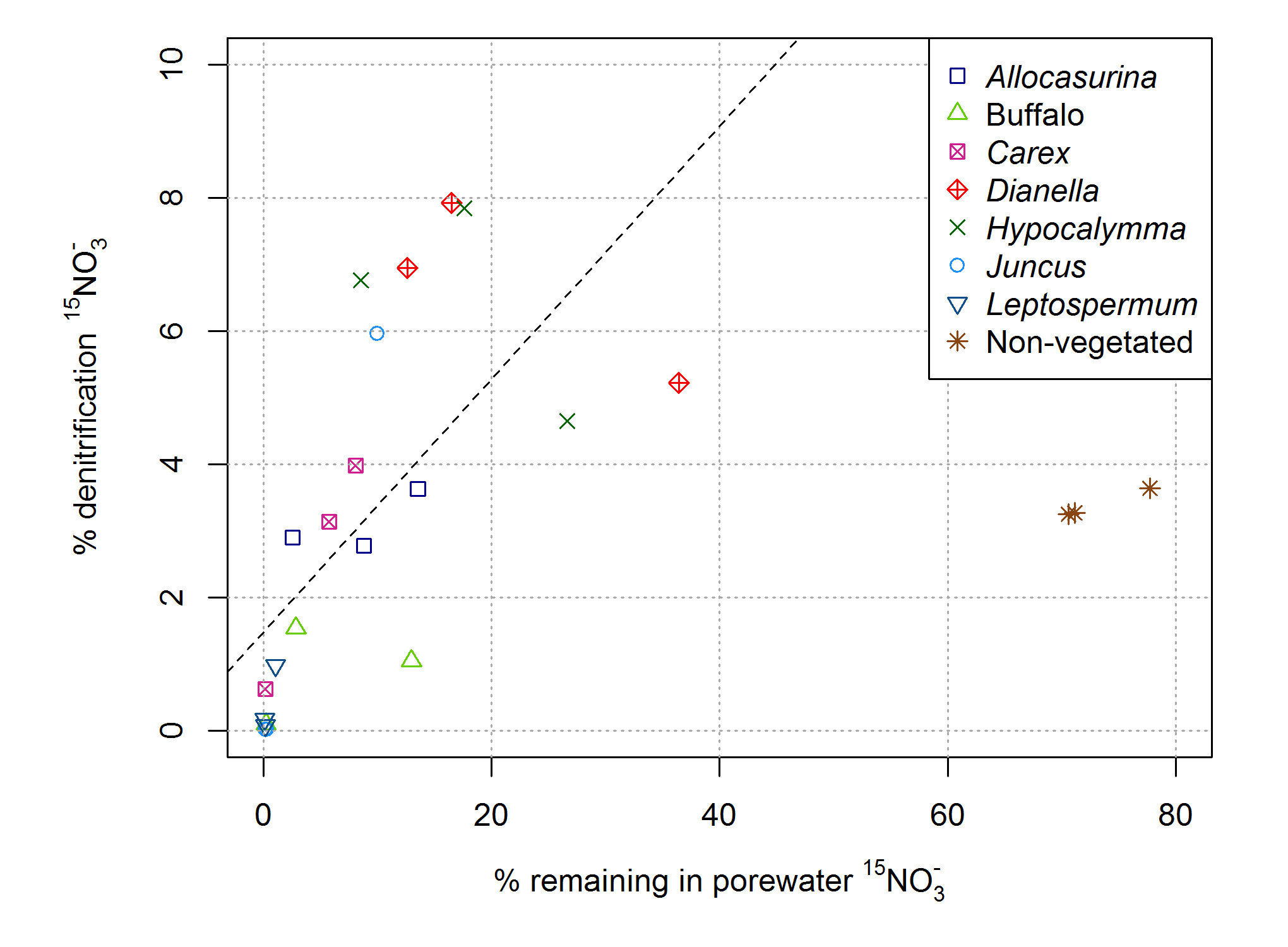

Supplement: Figure S2 — Relationship between percentage of 15NO3− remaining in the porewater and denitrified. Results for each biofilter column in the multiple species experiment (3 replicates per species, n = 1). Linear regression line fitted to the vegetated treatments only. (TIFF) [file pone.0090890.s002.tiff]

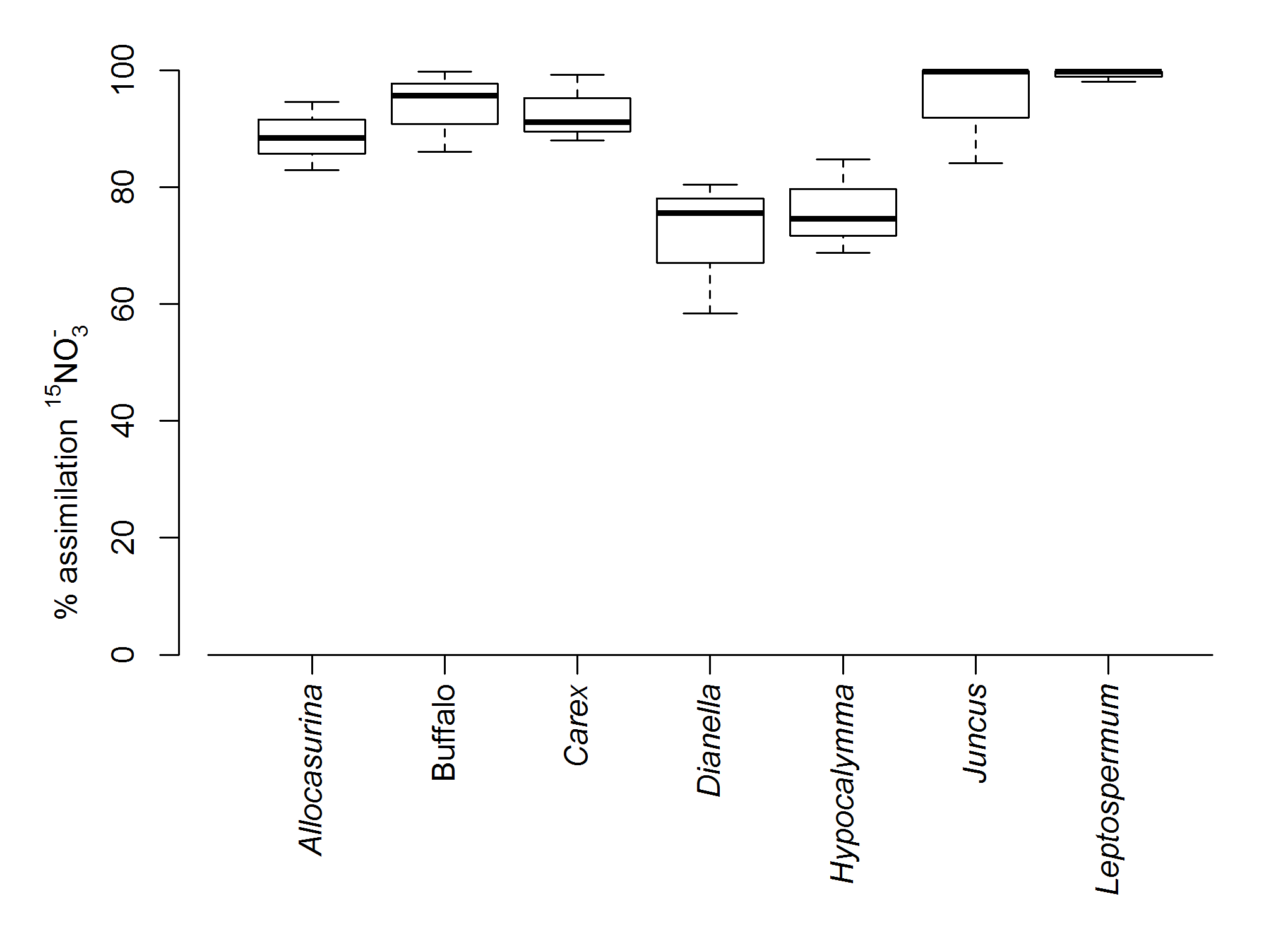

Supplement: Figure S3 — Boxplot comparison of percentage of 15NO3− assimilated across the vegetated treatments. Results for replicated columns in the multiple species experiment (3 replicates per species, n = 3). (TIFF) [file pone.0090890.s003.tiff]

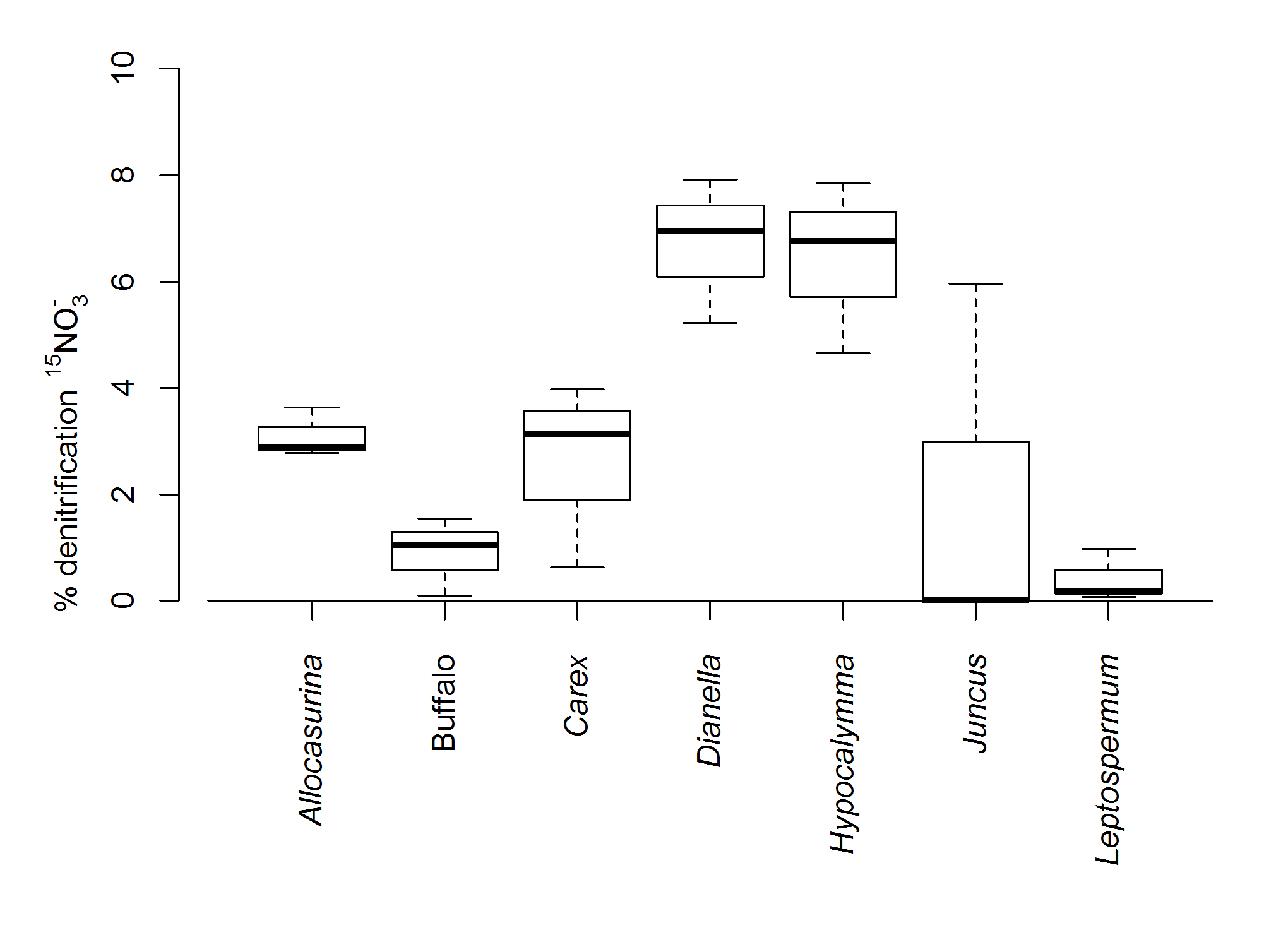

Supplement: Figure S4 — Boxplot comparison of percentage of 15NO3− denitrified across the vegetated treatments. Results for replicated columns in the multiple species experiment (3 replicates per species, n = 3). (TIFF) [file pone.0090890.s004.tiff]

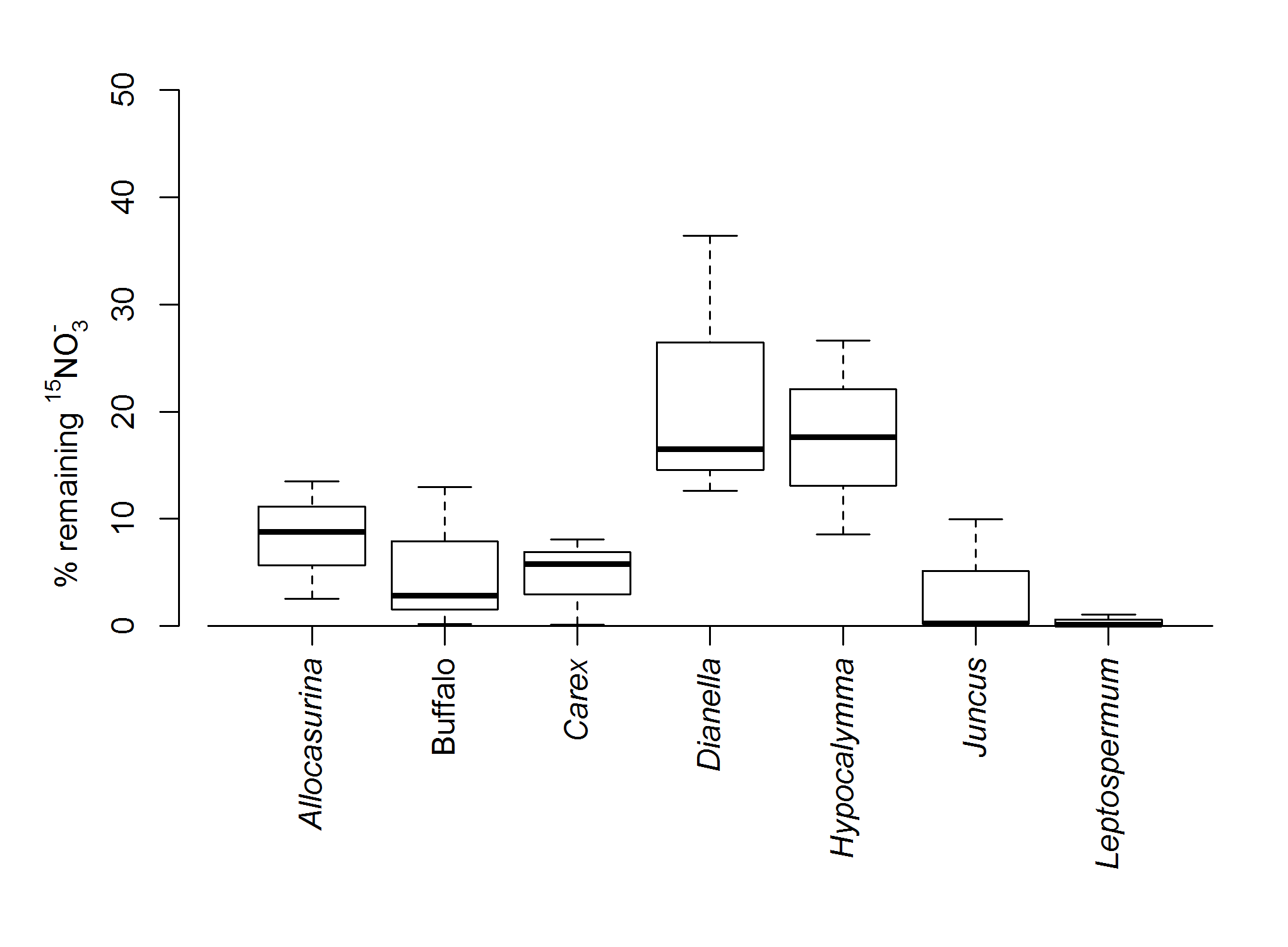

Supplement: Figure S5 — Boxplot comparison of percentage of 15NO3− remaining in the porewater across the vegetated treatments. Results for replicated columns in the multiple species experiment (3 replicates per species, n = 3). (TIFF) [file pone.0090890.s005.tiff]
